# *Trypanosoma vivax*, *T. congolense* “forest type” and *T. simiae*: prevalence in domestic animals of sleeping sickness foci of Cameroon

**DOI:** 10.1051/parasite/2011182171

**Published:** 2011-05-15

**Authors:** H. Nimpaye, F. Njiokou, T. Njine, G.R. Njitchouang, G. Cuny, S. Herder, T. Asonganyi, G. Simo

**Affiliations:** 1 Laboratoire de Biologie Générale, Département de Biologie et Physiologie Animales, Faculté des Sciences, Université de Yaoundé I BP 812 Yaoundé Cameroun; 2 Laboratoire de Recherche et de Coordination sur les Trypanosomoses IRD, UMR 177, CIRAD, TA 207/G, Campus International de Baillarguet 34398 Montpellier Cedex 5 France; 3 Faculty of Medicine and Biomedical Sciences, University of Yaoundé I Yaoundé Cameroun; 4 Department of Biochemistry, Faculty of Science, University of Dschang PO Box 67 Dschang Cameroun

**Keywords:** *T. vivax*, *T. congolense* “forest type”, *T. simiae*, PCR, pig, goat, sheep, dog, *T. vivax*, *T. congolense* “type forêt”, *T. simiae*, PCR, porc, chèvre, mouton, chien

## Abstract

In order to better understand the epidemiology of Human and Animal trypanosomiasis that occur together in sleeping sickness foci, a study of prevalences of animal parasites (*Trypanosoma vivax*, *T. congolense* “forest type”, and *T. simiae*) infections was conducted on domestic animals to complete the previous work carried on *T. brucei gambiense* prevalence using the same animal sample. 875 domestic animals, including 307 pigs, 264 goats, 267 sheep and 37 dogs were sampled in the sleeping sickness foci of Bipindi, Campo, Doumé and Fontem in Cameroon. The polymerase chain reaction (PCR) based method was used to identify these trypanosome species. A total of 237 (27.08%) domestic animals were infected by at least one trypanosome species. The prevalence of *T. vivax*, *T. congolense* “forest type” and *T. simiae* were 20.91%, 11.42% and 0.34% respectively. The prevalences of *T. vivax* and *T. congolense* “forest type” differed significantly between the animal species and between the foci (p < 0.0001); however, these two trypanosomes were found in all animal species as well as in all the foci subjected to the study. The high prevalences of *T. vivax* and *T. congolense* “forest type” in Bipindi and Fontem-Center indicate their intense transmission in these foci.

## Abstract (fr)

Afin de mieux comprendre l’épidémiologie des trypanosomoses humaines et animales sévissant ensemble dans les foyers de maladie de sommeil, une étude des prévalences des infections par *Trypanosoma vivax*, *T. congolense* “type forêt” et *T. simiae* a été menée chez les animaux domestiques pour compléter celle ayant porté sur la prévalence de *T. b. gambiense*. L’étude a concerné 875 animaux domestiques dont 307 porcs, 264 chèvres, 267 moutons et 37 chiens échantillonnés dans les foyers de maladie du sommeil de Bipindi, Campo, Fontem et Doumé au Cameroun. L’identification spécifique de ces trypanosomes a utilisée la technique de PCR. Au total, 237 (27,08 %) animaux domestiques étaient infectés par au moins une espèce de trypanosome. Les prévalences de *T. vivax*, *T. congolense* “type forêt” et *T. simiae* étaient de 20,91 %, 11,42 % et 0,34 % respectivement. Les prévalences de *T. vivax* et *T. congolense* “type forêt” diffèrent significativement entre les espèces animales et entre les foyers (p < 0,0001); toutefois, ces deux trypanosomes ont été identifiés chez les quatre espèces animales et dans tous les foyers soumis à l’étude. Les prévalences les plus élevées de *T. vivax* et *T. congolense* “type forêt” à Bipindi et Fontem-Centre indiquent une transmission intense de ces trypanosomes dans ces foyers.

## Introduction

Animal trypanosomosis constitutes a serious handicap to animal husbandry in all regions of sub- Saharan Africa infested by tsetse flies (Vaucel *et al.*, 1963). The infection agents are protozoans of the genus *Trypanosoma*. In tsetse infected areas, species and subspecies *T. congolense*, *T. vivax*, *T. simiae* and to a less extend *T. b. brucei* are pathogenic parasites to animals, especially Suidae and domestic ruminants (Omeke, 1994; Reifenberg *et al.*, 1997). Most of these trypanosome species pathogenic to animals are transmitted by tsetse flies. However, *T. vivax* can be mechanically transmitted by Tabanids or Stomoxys in Africa, and it is presumed to be transmitted only mechanically in areas not infested by tsetse flies (Gardiner & Wilson, 1987; Gardiner, 1989; D’Amico *et al.*, 1996). Moreover, experimental studies have demonstrated the possibility of mechanical transmission of *T. congolense* and *T. brucei* species by tabanids and *Stomoxys* (Mihok *et al.*, 1995; Sumba *et al.*, 1998). However, the importance of such transmission under natural conditions is still under debate (Desquesnes & Dia, 2003).

In animals, infection with trypanosome may result in a chronic, debilitating, emaciating and often fatal disease but the outcome of the infection differs substantially between trypanosome species or subspecies, between livestock species and within a livestock species among breeds depending on the challenge and virulence of the strains (Connor & van Den Bossche, 2004). Due to their frequencies, pathogenicity and consequence on productivity, *T. congolense* and *T. vivax* are the principal trypanosomes in domestic ruminants (Wellde *et al.*, 1983; Trail *et al.*, 1991).

Desoxyribonucleic acids (DNA) probes have allowed the identification of four different sub-species of *T. congolense* in different ecological zones: *T. congolense* “forest type”, *T. congolense* “savannah type”, *T. congolense* “Kilifi type” and *T. congolense* “Tsavo” (Majiwa *et al.*, 1985; Masiga *et al.*, 1992; Clausen *et al.*, 1998; Majiwa *et al.*, 1993). These variants of *T. congolense* are pathogenic and develop high parasitaemia accompanied by anemia and leucopenia (Sidibe *et al.*, 2002; Bengaly *et al.*, 2002a, b). However, there are clear differences in the pathogenicity among the various types of *T. congolense* and even within one single type. For example, experimental studies comparing the virulence of one strain of each subgroup in mice and cattle have shown differences between the subgroups with the *T. congolense* strain of the Savannah subgroup being the most virulent (Bengaly *et al.*, 2002a, b). Moreover, substantial differences in the virulence of *T. congolense* strains of the “savannah” subgroup, isolated in one geographical area from a single host species have also been recently reported (Masumu *et al.*, 2006).

For *T. vivax* infections, it is well known that animals infected by this trypanosome support better the infection because their genetic diversity is more limited than that of *T. congolense* and *T. brucei* (Authié *et al.*, 1999). However, fulminant and hemorrhagic forms of *T. vivax* that cause death or abortion have been described (Hudson, 1944; Olubayo *et al*, 1985). *T. congolense* and *T. vivax* species have been shown to cause serious infections in horses and asses (Faye *et al.*, 2001; Dhollander *et al.*, 2006). *T. simiae* has been reported as a parasite of suidae (Stephen, 1966).

During herd control by veterinary services, *T. vivax*, *T. congolense* or *T. b. brucei* were found in pigs, ruminants and equines in West Africa (Mattioli *et al.*, 1994; Omeke et al., 1994; Solano *et al.*, 1999; Faye *et al.*, 2001; Dhollander *et al.*, 2006). In the Central African region, limited work has been conducted on animal trypanosomosis, especially in sleeping sickness foci where *Trypanosoma brucei gambiense* is the causative agent of the disease. Indeed, in the forest area of southern Cameroon where studies were undertaken in the historic sleeping sickness foci of Fontem and Mbam, *T. congolense*, *T. vivax* and *T. simiae* were identified in pigs and small domestic ruminants (Asonganyi *et al.*, 1986; Asonganyi *et al.*, 1990; Simo *et al.*, 2006). Moreover, the presence of *T. brucei* s.l., *T. congolense* and *T. vivax* were reported in cattle of the pastoral zone of Adamaoua (North-Cameroon) (Mamadou *et al.*, 2006). However, most of these works were performed either on one species of domestic animals or used parasitological and immunological methods. Given the low sensitivity and specificity of these methods, it is obvious that the prevalence of different trypanosomes species or subspecies were considerably underestimated. With the development of molecular biology during the last decades, specific DNA sequences of different trypanosome species and subspecies have been identified and several PCR based methods were developed to improve the detection of various parasites. Applied in human and animal trypanosomosis, PCR appeared as a reliable, sensitive and specific techniques enabling to detect different trypanosome species and subspecies in vertebrate hosts as well as in tsetse flies (Masiga *et al.*, 1992; Desquesnes & Tresse, 1996; Penchenier *et al.*, 2000; Mugutu *et al.*, 2001; Picozzi *et al.*, 2002; Geysen *et al.*, 2003; Delespaux *et al.*, 2003; Gonzales *et al.*, 2005; Cox *et al.*, 2005).

The present study reports the level of infections with *T. vivax*, *T. congolense* “forest type” and *T. simiae* in four species of domestic animals commonly found in sleeping sickness foci of forest areas of southern Cameroon.

## Materials and Methods

### Study area

**Fig. 1.**
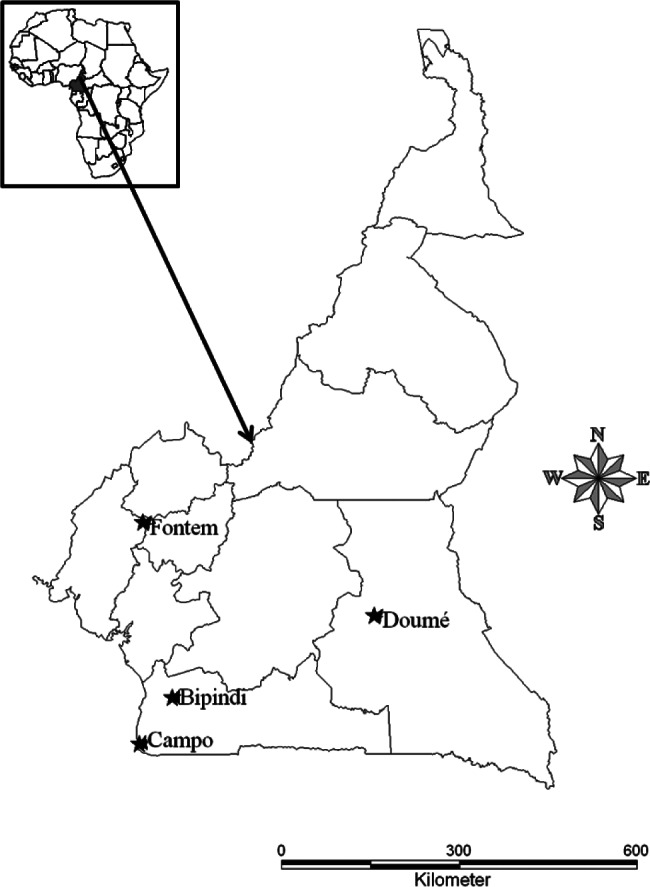
Study area in Cameroon. M: Molecular weight markers (100 bp); C + : *T. vivax* DNA (150 bp); C-: negative control; 1-9 samples; 2; 4; 7 and 8: negative samples; 1; 3; 5; 6 and 9: positive samples.

The study was carried from 2002 to 2004 in four sleeping sickness foci of the forest region of Southern-Cameroon (Fig. 1):

- Bipindi (3°2’N, 10°22’E), is a historic sleeping sickness focus discovered in 1920 which has been in recrudescence recently (Grébaut *et al.*, 2001). It lies between Lolodorf and Kribi, at 75 Km from the Atlantic Ocean. Though, *Glossina palpalis palpalis* is the dominant tsetse fly species, other tsetse fly species like *G. pallicera*, *G. caliginea* and *G. nigrofusca* can be also found in this focus (Morlais *et al.*, 1998; Simo *et al.*, 2008). The domestic animals were bled in the villages Bijouka, Lambi, Memel, Bipindi-Centre and Ebimingbang.
- Campo (2°20’N, 9°52’E), is a hypo-endemic focus where a few sleeping sickness patients are detected each year. It lies along the River Ntem which separates Cameroon and Equatorial Guinea and flows into the Atlantic Ocean. Several tsetse fly species including *G. p. palpalis*, and to a lesser extent, *G. pallicera*, *G. caliginea* and *G. nigrofusca* are encountered in this sleeping sickness focus (Morlais *et al.*, 1998; Simo *et al.*, 2008). Samples were taken from domestic animals in the villages Akak, Mabiogo, Ipono, and Campo center.
- Fontem (5°40’N, 9°55’E) is a sleeping sickness focus known since 1949 where the prevalence of sleeping sickness has reduced considerably. It has a much varied topography with numerous hills and valleys through which high speed rivers flow in the South Western Region of Cameroon. *G. p. palpalis* is the only tsetse fly species found in this area (Morlais *et al.*, 1998; Simo *et al.*, 2008). The focus is divided into three sub-foci (North, Center and South). In the Center sub-focus where the samples were taken (villages of Menji, Fotabong, Soko, Azi), few sleeping sickness patients were detected during the last decade. In the Northern villages (Bechati, Folepi, Besali), no patients have been detected during the last 20 years (Ebo’o Eyenga, personal communication), although in pigs, the prevalence of *T. b. gambiense* group 1 infections was 15% in 1999 (Nkinin *et al.*, 2002).
- Doumé (4°16’N, 13°25’E) is an old sleeping sickness focus in the Eastern Region of Cameroon where very few sleeping sickness patients were detected during the last decade. *Glossina fuscipes* is the main tsetse fly species found in this focus (Mbida Mbida *et al.*, 2009). Doumé is a degraded forest zone with many rivers and vast areas of wetlands. Samples were collected from domestic animals in the villages Medim, Paki, Baillon and Loumbou.

In these four sleeping sickness foci where cattle is rare, pigs, sheep, goats and chickens are kept to meet dietary, ceremonial and commercial needs. Dogs serve as pets and hunting companions.

### Blood collection from animals

Domestic animals were sampled during five field surveys: one was done at Doumé (October 2002), one at Bipindi (July 2003), one at Fontem (October, 2003) and two at Campo (April 2003 and June 2004). During each survey, about one in three animals that has spent at least three months in the focus was sampled and bled with the cooperation of the owners, but some dogs did not cooperate. Goats and sheep were bled from the jugular vein; pigs from the sub-clavicular vein and dogs from the cephalic vein. The blood was put in EDTA coated tubes, labeled and preserved at 4 °C for molecular analyses.

All pigs and dogs sampled in this study were of a local breed, originating from a mixture of different breeds. The sheep and goats are Dwarf breeds (Djallonke West-African Dwarf for sheep and Guinea goat), which are known to be trypanotolerant.

### Extraction and amplification of DNA

DNA was extracted from the samples using the kit “Ready Amp Genomic DNA purification system” (PROMEGA) essentially as described by Penchenier *et al.*, (2000). The supernatant containing DNA was stored at -20 °C or used directly for PCR.

Amplification of trypanosome DNA was conducted using the primer pairs TCF1 (GGACACGCCAGAAGGTACTT) / TCF2 (GTTCTCGCACCAAATCCAAC), TVW1 (CTGAGTGCTCCATGTCCCAC) / TVW2 (CCACCAGAACACCAACCTGA), and TSM1 (CCGGTCAAAAACGCATT) / TSM2 (AGTCGCCCGGAGTCGAT) specific for *T. congolense* “forest type”, *T. vivax* and *T. simiae* respectively (Masiga *et al.*, 1992). The amplifications were conducted in a total volume of 25 μl containing 2.5 μl of PCR buffer 10X [10 mM Tris – HCl (pH 9.0), 50 mM KCl, 3 mM MgCl_2_], 15 picomoles of each primer, 200 μl of deoxynucleotide-triphosphate (dNTP), one unit of Taq DNA polymerase (Appligene-Oncor, USA), sterile water and 3 μl DNA extract. Amplification involved pre-denaturation at 94 °C for 3 min 30 s followed by 40 cycles of denaturation at 94 °C (30 s), hybridization of primers at 60 °C and elongation at 72 °C for 1 minute, then final elongation at 72 °C for 5 min. The amplification products were resolved on 2% agarose gel containing ethidium bromide (0.3 μg/ml). DNA bands were visualized under ultraviolet (UV) light.

### Statistical analyses

The proportions of animals infected by different trypanosome species were compared between animals and localities using Chi-square (χ^2^) test of the Statistix Computer program.

## Results

**Fig. 2.**
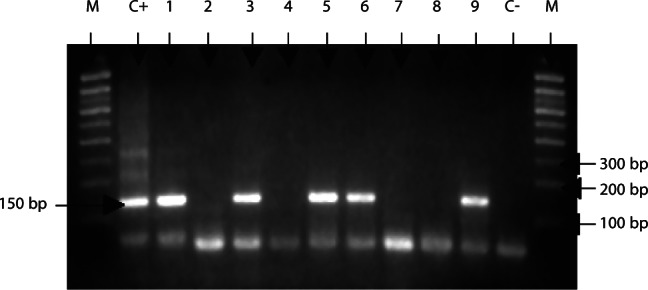
Electrophoretic profile showing the resolution of *T. vivax* specific DNA.

Fig. 2 shows an example of profiles obtained after resolution of PCR products from the amplification of *T. vivax* DNA. Tables I and II report the number of animals analyzed and the PCR-positive samples by animal species and localities. Although only the results of three trypanosomes species (*T. vivax*, *T. congolense* “forest type” and *T. simiae*) are reported in this study, *T. brucei* s.l. and *T. b. gambiense* group 1 (human infective trypanosome) were also investigated and the results are reported in Njiokou *et al.* (2010). These authors showed that 19.88% and 3.08% of the animals were infected by *T. brucei* s.l. and *T. b. gambiense* respectively. *T. b. gambiense* were harboured by pigs, already known to be reservoir hosts, but also by goats and sheep, pointing out their contribution to the epidemiology of HAT. The prevalences significantly varied according to the animal species and the focus, in connection with the level of endemicity of HAT. In this study, 27.08% (237/875) of animals analyzed were infected by at least one of the three trypanosome species (Tables I and II). The levels of infection differed significantly between animal species (χ^2^ = 77.92; p < 0.0001) and between localities (χ^2^ = 52.89; p < 0.0001). *T. vivax* was the most predominant trypanosome in animals with a global infection rate reaching 20.91% and more precisely 36.15% in pigs, 18.18% in goats, 8.61% in sheep and 2.7% in dogs. *T. congolense* “forest type” was identified in 11.42% of the animals, and 19.86%, 10.22%, 8.1% and 3.37% for pigs, goats, dogs and sheep respectively. *T. simiae* was rare (0.34%) and was diagnosed only in two (0.75%) goats and one (0.37%) sheep. The frequency of *T. vivax* and *T. congolense* “forest type” differed significantly between animal species and between localities (p < 0.0001). The comparison of *T. vivax* infection rates in different pairs of animal species showed significant differences, except for the sheep/dog pair. Significant differences were also observed for the *T. congolense* “forest type” infection rates for the pig/goat, pig/sheep and goat/sheep pairs.

**Table I.** Number and percentage of animals infected with *T. congolense*, *T. vivax* and *T. simiae*.

	Number of positive PCR (percentage)				
Animal species	NE	TCF	TVW	TSM	NP
Pigs	307	61 (19.86)	111 (36.15)	0 (0)	133 (43.32)
Goats	264	27 (10.22)	48 (18.18)	2 (0.75)	69 (26.13)
Sheep	267	9 (3.37)	23 (8.61)	1 (0.37)	31 (11.61)
Dogs	37	3 (8.1)	1 (2.7)	0 (0)	4 (10.81)
Total	875	100 (11.42)	183 (20.91)	3 (0.34)	237 (27.08)

χ^2^		39,5	76,15		77,92
p		0.0001	0.0001		0.0001

The number of positive PCR are given with their percentage in the brackets; TCF: *Trypanosoma congolense* “forest type”; TVW: *Trypanosoma vivax*, TSM: *Trypanosoma simiae*; NE: number of animals examined; NP: number of animals parasite-positive (having DNA of at least one species), χ^2^: Chi-square test; p: p value.

**Table II.** Number and percentage of animals infected with *T. congolense*, *T. vivax* and *T. simiae* in various localities.

	Number of positive PCR (percentage)				
Locality	NE	TCF	TVW	TSM	NP
Bipindi	204	30 (14.7)	65 (31.86)	0 (0)	80 (39.21)
Campo	310	32 (10.32)	65 (20.96)	2 (0.64)	82 (26.45)
Fontem-center	154	28 (18.18)	44 (28.57)	0 (0)	55 (35.71)
Doumé	207	10 (4.83)	9 (4.34)	1 (0.48)	20 (9.66)
Total	875	100 (11.42)	183 (20.91)	3 (0.34)	237 (27.08)

χ^2^		18.3	54.6		52.89
p		0.0001	0.0001		0.0001

The number of positive PCR are given with their percentage in the brackets; TCF: *Trypanosoma congolense* “forest type”; TVW: *Trypanosoma vivax*, TSM: *Trypanosoma simiae*; NE: number of animals examined; NP: number of animals parasite-positive (having DNA of at least one species), χ^2^: Chi-square test; p: p value.

The comparison by locality showed that animals from Bipindi and Fontem-Center are more frequently infected with *T. vivax* and *T. congolense* “forest type”. Howerver, the level of infection of these trypanosome species does not differ significantly between these two localities (χ^2^ = 0.44; p = 0.5) and (χ^2^ = 0.78; p = 0.37) respectively. Animals from Doumé were significantly less infected by *T. vivax* and *T. congolense* “forest type” than those of the other localities. Significant differences were observed between the *T. vivax* infection rates in Bipindi and Campo (χ^2^ = 7.72; p = 0.005) as well as between the *T. congolense* “forest type” infection rates in Fontem-Center and Campo (χ^2^ = 5.64; p = 0.017). However, animals from Bipindi and Fontem-Center are more infected by *T. vivax* and *T. congolense* “forest type” respectively.

Mixed infections were found in 32 animals including 26 pigs and 6 goats carrying both *T. congolense* “forest type” and *T. vivax*; one sheep carried both *T. vivax* and *T. simiae*; and one goat carried triple infections of *T. congolense* “forest type”, *T. vivax* and *T. simiae* (Table III). When taking into account the occurrence of *T. b. gambiense* revealed in Njiokou *et al.*, (2010), three other double infections with *T. b. gambiense* group 1 and *T. vivax* are identified in one sheep and one goat while *T. b. gambiense* group 1 and *T. congolense* “forest type” was identified in one sheep. Furthermore, one goat was infected with four trypanosomes including *T. b. gambiense* group 1, *T. vivax*, *T. congolense* “forest type” and *T. simiae*.

**Table III.** Type, nature and number of infections revealed by PCR in different domestic animal species.

		Number of single and mixed infections				
Type of infections	Trypanosome identified	Pigs	Goats	Sheep	Dogs	Total
Single	TCF	70	31	11	1	113
	TVW	19	17	6	2	44
	TSM	0	1	0	0	1

Double	TCF et TVW	26	6	0	0	32
	TVW et TSM	0	0	1	0	1

Triple	TCF, TVW et TSM	0	1	0	0	1

TCF: *Trypanosoma congolense* “forest type”; TVW: *Trypanosoma vivax*, TSM: *Trypanosoma simiae*.

## Discussion

In this study, PCR revealed infections with *T. congolense* “forest type”, *T. vivax* and *T. simiae* in the domestic animals, thus confirming the circulation of these parasites in the sleeping sickness foci of southern Cameroon as previously reported in tsetse flies (Morlais *et al.*, 1998), wild animals (Herder *et al.*, 2002; Njiokou *et al.*, 2004; Njiokou *et al.*, 2006) and pigs (Penchenier *et al.*, 1996; Simo *et al.*, 2006). The infection rate of 27.08% is in line with results obtained in pigs, small domestic ruminants, and dogs in the Central and West African regions (Asonganyi *et al.*, 1986, 1990; Makumyaviri *et al.*, 1989; Omeke, 1994; Penchenier *et al.*, 1996; Dadah *et al.*, 1997; Kalu *et al.*, 2001), also out of any sleeping sickness areas.

The high prevalence of *T. vivax* corroborates other findings in domestic animals (Bourzat & Gouteux, 1990; Dadah *et al.*, 1997; Kalu *et al.*, 2001) as well as wild animals (Komoin-Oka *et al.*, 1994; Truc *et al.*, 1997; Herder *et al.*, 2002; Njiokou *et al.*, 2004; Njiokou *et al.*, 2006) of the Central and West Africa regions. This high prevalence of *T. vivax* may result from the level of pathogenicity of this trypanosome, which is generally low and better controlled by animals (Stephen, 1970; Authié *et al.*, 1999). It may result also from the mechanical transmission, which has not been reported in the other species studied here, except in certain extend *T. congolense*.

The identification of *T. vivax* in pigs is in accord with results obtained by Ng’ayo *et al.* (2005) in East Africa. Using specific primers, these authors showed that *T. vivax* infections are frequent in pigs and goats. Penchenier *et al.* (1996) and Simo *et al.* (2006) also found numerous *T. vivax* infections in pigs from Cameroon, by PCR. These results need to be confirmed by other techniques because it is generally accepted that pigs are refractory to infections with *T. vivax* (Taylor & Authié, 2004). The fact that *T. vivax* is detected only by PCR based methods and not by parasitological techniques could be explained by ‘‘transient’’ infections. Moreover, with the development of new genotyping tools that do not require isolation of trypanosomes, it is urgent to characterize *T. vivax* circulating in pigs as well as in other vertebrate hosts in order to investigate if some strains might be infective to pigs and others non-infective. Indeed, previous studies have suggested genetic variation in *T. vivax* populations that renders some isolates more pathogenic than others (Mwongela *et al.*, 1981; Wellde *et al.*, 1983; Roeder *et al.*, 1984).

The lower prevalence of *T. congolense* “forest type” with respect to *T. vivax* in domestic animals may result from higher parasitemia in *T. congolense* infections, accompanied by serious anemia, which leads to the rapid death of the host animal (Sidibe *et al.*, 2002; Bengaly *et al.*, 2002a, b).

The very low prevalence of *T. simiae* as already reported in pigs (Penchenier *et al.*, 1996; Simo *et al.*, 2006) and wild animals (Herder *et al.*, 2002; Njiokou *et al.*, 2004) of several sleeping sickness foci indicates a low transmission of this parasite in various localities of Cameroon. The absence of *T. simiae* in pigs of various areas of Cameroon is likely due to its high pathogenicity because pigs infected with this trypanosome species would probably not survive. Our results corroborate those obtained by Simo *et al.* (2006) in pigs of the Fontem sleeping sickness focus of Cameroon.

Our results also showed a significant difference in the prevalence of *T. vivax* and *T. congolense* “forest type” between the different animal species. Pigs are more infected than goats, sheep and dogs; this could either be indicative of a higher susceptibility of pigs to *T. vivax* and *T. congolense*, or of a higher frequency of contact with the tsetse fly vector. Indeed, it has been shown that *G. p. palpalis* takes more blood meals from pigs than from goats and sheep and rarely on carnivores (Dagnogo *et al.*, 1985; Sané *et al.*, 2000; Späth, 2000; Simo *et al.*, 2008). The keeping of pigs in sties near habitations exposes them to more contacts with peri-domestic tsetse flies (Frézil *et al.*, 1980) than animals that roam freely. Finally, the highest prevalences of *T. vivax* and *T. congolense* “forest type” in domestic animals from Bipindi and Fontem-Centre respectively, confirm the results reported in wild animals from Bipindi (Herder *et al.*, 2002; Njiokou *et al.*, 2004) and in domestic animals from Fontem (Asonganyi *et al.*, 1990) and indicate their intense transmission in these sleeping sickness foci.

The presence of *T. vivax* and *T. congolense* in all the localities studied is not only indicative of the ubiquity of the trypanosomes, but also of the presence of an appropriate vector in the foci. Their prevalence in Bipindi and Fontem where *G. p. palpalis* represents about 100% is higher than in Campo where *G. p. palpalis* represents only 56% and Doumé where *G. fuscipes* is the only tsetse fly species found (Mbida Mbida *et al.*, 2009).

Our results on mixed infections in pigs corroborate those of Jamonneau *et al.*, (2004) who reported a high proportion of mixed infection in pigs of the Bonon sleeping sickness focus in Côte d’Ivoire and those of Simo *et al.*, (2006) in pigs of the Fontem sleeping sickness focus in Cameroon. These results are in line with those obtained in tsetse flies (Morlais *et al.*, 1998; Masiga *et al.*, 1996; Reifenberg *et al.*, 1997; Malele *et al.*, 2003). Indeed, entomological studies showed that infected tsetse flies from the sleeping sickness foci of Cameroon (Morlais *et al.*, 1998) and other zones of Africa (Masiga *et al.*, 1996; Reifenberg *et al.*, 1997; Malele *et al.*, 2003) frequently harbour more than one trypanosome species. This demonstrates the high probability of tsetse flies to transmit several trypanosome species to vertebrate hosts. Up till now, the interaction and the evolution of different trypanosome species or subspecies in the infected hosts remain not yet well understood. Experimental studies have shown that primary infection with *T. congolense* prevents the establishment of a second strain of *T. congolense* (Morrison *et al.*, 1982) while an animal already infected with *T. congolense* becomes refractory to *T. brucei* s.l. infections (second infection), but not *T. vivax* (Dwinger *et al.*, 1989). Results of our study, especially the low proportion of mixed infection including *T. congolense* “forest type” and *T. b. gambiense* (see also Njiokou *et al.*, 2010 data) and the considerable numbers of mixed infections involving *T. vivax* and *T. congolense* “forest type” play in favor of these experimental studies. However, in the field conditions where tsetse flies are infected by several trypanosome species, it is obvious that several trypanosome species or subspecies are transmitted to the same hosts. In such conditions, the prevalence of each species of trypanosome in the mixed infections may reflect probably the prevalence of this trypanosome species in the locality. Our results on the mixed infections of several trypanosomes species in vertebrate hosts suggest further investigations on the establishment and the evolution of different trypanosomes species or subspecies in the vertebrate hosts. Such investigations may enable to understand some aspects of the Human African Trypanosomiasis as well as Animal African Trypanosomiasis. For example, these investigations may enable to know if some trypanosome species can prevent the establishment and the evolution of *T. b. gambiense* and if genetic exchanges between the same trypanosome species can occur in vertebrate hosts.
